# ERG induces a mesenchymal-like state associated with chemoresistance in leukemia cells

**DOI:** 10.18632/oncotarget.1449

**Published:** 2013-12-04

**Authors:** Liliana H. Mochmann, Martin Neumann, Eva K. von der Heide, Verena Nowak, Anja A. Kühl, Jutta Ortiz-Tanchez, Juliane Bock, Wolf K. Hofmann, Claudia D. Baldus

**Affiliations:** ^1^ Department of Hematology and Oncology, Charité University Medicine Berlin, Berlin, Germany; ^2^ Department of Hematology and Oncology, University Hospital Mannheim, Mannheim, Germany; ^3^ Department of Gastroenterology, Infectiology and Rheumatology, Charité University Medicine Berlin, Berlin, Germany

**Keywords:** ERG, ERK, EMT, Chemoresistance

## Abstract

Overexpression of the oncogene ERG (ETS-related gene) is an adverse prognostic factor in acute myeloid and T-cell lymphoblastic leukemia (AML and T-ALL). We hypothesize that ERG overexpression is associated with primary drug resistance thereby influencing the outcome in leukemia. We previously reported a cell-line based model of ERG overexpression which induced a potentially chemo-resistant spindle shape cell type. Herein, we report a specific transcriptional gene signature for the observed spindle shaped morphology. Genes significantly over-expressed after ERG induction strongly resembled adhesive mesenchymal-like genes that included integrins (ITGA10, ITGB5, ITGB3, ITGA2B), CD44, and CD24. Interestingly, the mesenchymal-like signature was accompanied by the repression of DNA chromatin remodeling and DNA repair genes, such as CHEK1, EZH2, SUZ12, and DNMT3a. The ERG-induced mesenchymal-like signature positively correlated with TMPRSS2-ERG prostate tissues and invasive breast cancer mRNA expression datasets reflecting a general ERG-driven pattern of malignancy. Furthermore, inhibitors modulating ERG druggable pathways WNT, PKC, and AKT, and chemotherapeutic agent cytarabine revealed ERG-induced drug resistance. In particular, PKC412 treatment enhanced proliferative rates and promoted spindle shape formation in ERG-induced cells. Nilotinib and dasatinib were effective at abolishing ERG-induced cells. Moreover, ERG overexpression also led to an increase in double strand breaks. This report provides mechanistic clues into ERG-driven drug resistance in the poor prognostic group of high ERG expressers, provides insight to improved drug targeted therapies, and provides novel markers for a mesenchymal-like state in acute leukemia.

## INTRODUCTION

The oncogene ERG belongs to an evolutionary related group of ETS DNA binding proteins and directs gene expression in hematopoietic processes establishing definitive hematopoiesis, maintaining the stem cell pool[1] and promoting megakaryocytic differentiation[2]. Chromosomal aberrations harboring a fusion product of ERG to form FUS/TLS-ERG in acute myeloid leukemia (AML)[3], ERG-EWS in Ewing's sarcoma[4], or TMPRSS2-ERG[5,6] in prostate cancers are predictive of poor prognosis. Likewise, high levels of ERG correlate with a worse outcome in cytogenetically normal AML and acute T-lymphoblastic leukemia (T-ALL)[7,8]. Mouse models overexpressing Erg clearly revealed an oncogenic phenotype, with high Erg causing fetal hematopoietic progenitors to develop leukemia[2]. Similarly, high ERG expressing bone marrow cells transplanted in adult mice produced Notchl mutations and T cell expansion[9]. Recently it was reported that about 30% of transgenic ERG mouse models develop T-ALL[10] whereas the remainder develop myeloid leukemia at five months[11].

Current chemotherapy regimens are insufficient for high-risk acute leukemia patients characterized by high ERG expression. For instance, in AML, the cumulative incidence of relapse in high ERG expressers was 81%, in comparison to only 33% in low ERG expressers at 5-years[7]. Similarly, the overall survival of high ERG expressers in T-ALL at 5 year years is only 26% versus 58% in low ERG expressers[8]. Thus, understanding the ERG gene regulatory networks responsible for treatment failure and involved in drug resistance at the molecular level will aid in understanding the etiology of high ERG expression in acute leukemia.

Due to the high incidence of TMPRSS2-ERG fusion in prostate cancer, recent studies have mainly focused on mapping ERG signaling networks in prostate. These networks comprise a diaspora of functions that show a role for ERG in the regulation of extracellular matrix through the plasminogen activator pathway[12], upregulation of epithelial-to-mesenchymal transition (EMT) genes[13], ERG-mediated regulation of chromatin though binding to the EZH2 promoter, and DNA repair regulation through poly (ADP-ribose) polymerase (PARP) interactions[14]. This composite ERG gene signatures correlates well with the clinical characteristics of prostate cancer, and is thought to contribute to disease progression in prostate cancer[15,16].

While it is unarguable that ERG overexpression is involved in oncogenesis of leukemia and prostate cancers, much less is clear as to how ERG signaling mediates drug resistance. Emerging reports describe EMT in tumor progression as a mechanism for cell proliferative and survival advantages[17]. EMT is defined as an epithelial cell undergoing transformation, acquiring mesenchymal-like features that allow a cell to be motile and able to migrate. This process requires specific changes in gene regulation and is remarkably reversible (termed mesenchyme-to-epithelial, MET) via epigenetic changes[18]. Moreover, the acquisition of mesenchyme-like (derived from MET) properties in both malignant cells and non-epithelial cells has been proposed as a mechanism for drug resistance in solid tumors of the lung, breast, prostate[18] and in chronic myeloid leukemia[17]. Several ETS transcription factors have been implicated in turning on an EMT-like program and, likewise, studies in cancer cells document enhanced cell migration in EMT overexpressing cells[19,20]. Taken together, these studies support the notion that EMT in high ERG expressers may contribute to drug resistance in prostate carcinoma. Herein, we report that in leukemia, ERG overexpression causes molecular characteristics that are strikingly similar to the ERG-associated signaling networks in prostate cancer. ERG overexpression induces a mesenchymal-like state with a highly drug resistant phenotype, pronounced proliferative growth advantage, and promotes double strand breaks (DSBs) formation. Our findings may have important clinical implications for the improvement of current therapies in adult leukemia.

## RESULTS

### ERG induction promotes mesenchymal-like gene expression signature accompanied by repression of DNA remodeling and DNA repair

**Figure 1:**
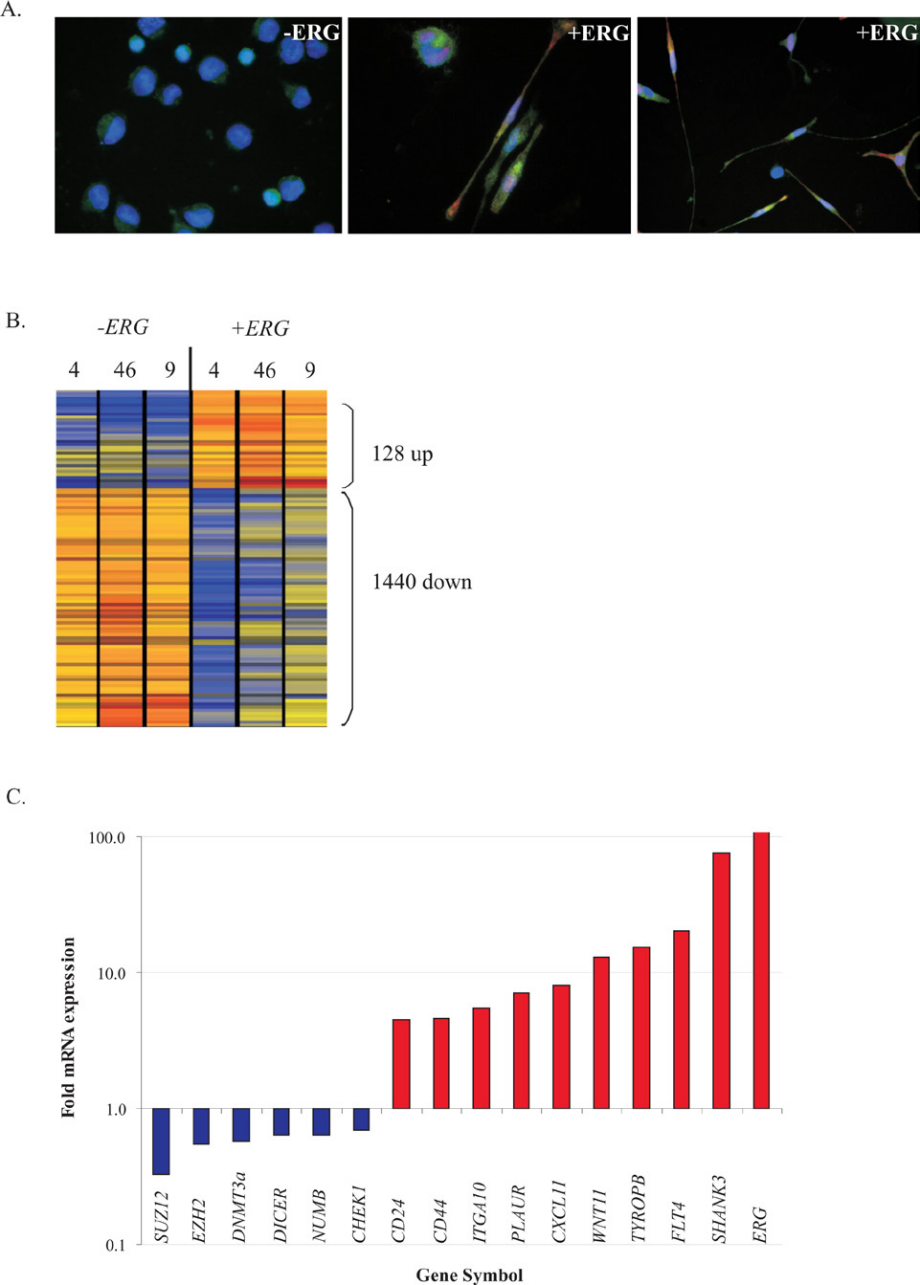
ERG overexpression induces a mesenchymal-like signature accompanied by repression of DNA homologous recombination repair gene expression A) Fluorescence imaging of ERG-induced cells (+ ERG, DsRed fluorescence) developing spindle shape formation following DOX stimulation for 96 hours. Rabbit anti-WNT11 antibody (1:100) coupled with goat anti-rabbit IgG-FITC (1:100) was used for colocalization with DsRed positive (+ERG). DAPI (1:100) was used to stain the nuclei. Non-induced (−ERG, left) maintain the native round morphology with basal levels of WNT11 on the cell surface. WNT11, a direct target of ERG, localizes to the nuclei and along the spindle branches (center, 400X magnifications and right, 200X magnification). B) Heat map displaying the ERG gene signature derived from three stable transfected K562 pTRE-ERG clones (CL4, CL46 and CL9, referred to as 4, 46 and 9) induced with DOX for 6 days. Red to orange hues, strong expression; blue to yellow hues, low expression of induced cells relative to non-induced cells. A 2-fold cut off was used to determine the significance of over-expressed (128) and under-expressed genes (1440). C) RT PCR validation of novel ERG transcriptional targets from Figure 1B related to kinase, DNA modification, or adhesion. Fold mRNA expression (y-axis) of each gene reports the ratio between induced and non-induced ERG mRNA expression. RT PCR was conducted in duplicate and bar graphs represent the data for the CL9 K562 pTRE-ERG clone.

We previously reported that prolonged ERG overexpression induced leukemia cells to adhere and develop bi-directional protrusions (spindle shaped cells) (Fig. 1A). This morphogenic state was in part attributable to the upreguation of WNT11[21]. However the overall transcriptional program for this morphogenesis had not been characterized (Fig 1A). Herein, we sought to elucidate the morphogenesis program responsible for the spindle shape formation upon ERG induction by determining the global transcription profile of leukemic K562 cells harbouring tet-on inducible ERG expression constructs. When comparing global gene expression of ERG-induced cells versus non-induced cells, 128 genes were differentially over-expressed whereas 1440 were differentially under-expressed (Figure 1B, fold change ≥2; p<0.05). Through Gene Ontology (GO) analyses, the over-expressed genes were significantly enriched for cell adhesion, migration, and motility (Table 1, top), a pattern that highly resembled the adhesive EMT properties and included integrin upregulation. We verified by PCR upregulation of gene products associated with biological adhesion and morphogenesis including: CD24, CD44, ITGA10, PLAUR CXCL11, SELP, TYROBP, FLT4, and SHANK3 (Figure 1C). While the EMT/MET genetic program has yet to be fully understood in cancer, recent studies showed that upregulation of two novel EMT key markers, CD44 and CD24, are necessary for entry into a mesenchymal-like state in highly drug resistant breast HER2 positive carcinoma[25]. Contrary to typical EMT/MET, the EMT markers E- and N-cadherin were not differentially altered by ERG overexpression, suggesting that the EMT/MET gene signature may be different in leukemia than in breast cancer. NUMB, both a binding target of ERG[26] and reported to be involved in EMT regulation[27], was also under-expressed (Figure 1C). Additional ERG under-expressed genes were enriched for the GO term “DNA homologous recombination and repair” including the CHEK1, ATM, and BRCA1 DNA repair genes (Table 1, bottom). DNA chromatin remodeling proteins, such as EZH2, SUZ12, and DNMT3a, were also under-expressed (Figure 1C), as observed in prostate cancer studies[14,28]. Hence, repression of DNA repair and DNA chromatin remodeling genes may provide a link to the molecular mechanisms through which ERG overexpressing cells induce a mesenchymal-like state.

Table 1ERG over expression results in a mesenchymal-like signature accompanied by dowreguation of DNA repairSignificantly over-expressed and under-expressed genes were uploaded and analyzed with DAVID Bioinformatics. Listed below are genes that signficantly cluster to the respective gene ontology functions. P-Value is included for significance.

**Table:** 

GOTERM (Over-expressed)	Count	P-Value	Gene Symbol
integrin-mediated signaling pathway	4	2.58E-03	*ITGA10, ITGB5, ITGB3, ITGA2B, FLT4*
Chemotaxis	5	3.52E-03	*CXCL1, SEMA3F, CXCL11, PLAUR, CMTM5*
response to wounding	8	4.40E-03	*CXCL1, AIF1, CLU, GP1BA, ITGB3, CXCL11, TIMP3, PLAUR*
cell-matrix adhesion	4	5.09E-03	*ITGA10, ITGB5, ITGB3, ITGA2B*
cell-substrate adhesion	4	6.65E-03	*ITGA10, ITGB5, ITGB3, ITGA2B*
immune response	8	1.74E-02	*CXCL1, LST1, CD300A, CLU, FCER1G, MYO1F, CXCL11, ARHGD1B*
cell adhesion	8	1.87E-02	*CD300A, PECAM1, GPR56, LTGA10, LTGB5, GP1BA, LTGB3, LTGA2B*
locomotory behavior	5	2.22E-02	*CXCL1, SEMA3F, CXCL11, PLAUR, CMTM5*
response to organic nitrogen	3	2.53E-02	*ALDOC, PTGS1, TLMP3*
regeneration	3	2.99E-02	*ALDOC, TIMP3, PLAUR*
wound healing	4	3.88E-02	*GP1BA, ITGB3, TLMP3, PLAUR*
leukocyte mediated immunity	3	4.47E-02	*CLU, FCER1G, MYOIF*

**Table:** 

BIOCARTA (Under-expressed)	Count	P-Value	Gene Symbol
Role of BRCA1, BRCA2 and ATR in Cancer	5	3.23E-02	*LOC651610, LOC648152, LOC651921, CHEK1, ATR, ATM, RAD50, BRCA1*

We then used Oncomine, a microarray cancer database, to assess whether ERG-induced gene signature generated from the K562 pTRE-ERG leukemia cell line was disease-relevant. 128 over-expressed genes were uploaded to Oncomine platform. ERG-induced gene signature positively correlated with three invasive and metastatic cancer mRNA expression dataseis: 1) Grasso dataset prostate tissues habouring ERG rearrangement versus no ERG rearrangement[29], 2) Grasso dataset of prostate carcinoma tissues versus normal prostate gland[29] and 3) Stickeler dataset of invasive breast cancer patients treated with a standard regimen of chemotherapy assessing post-chemotherapy versus pretreatment changes[30] (Supplement Figure 1A–D and Supplement Table 1). Of note, in the latter dataset, 17 of 25 patients with invasive breast cancer did not result in complete remission indicating that the analysis of transcriptionally deregulated genes could aid in predicting the clinical response to cancer treatment. Recent studies in a prostate cell line over-expressing ERG also concur with our results, i.e. they exhibit highly enriched cell adhesion and cell migration GO terms[31] and promote EMT gene expression[32], thus providing further support for an ERG-driven mesenchymal-like state in leukemia. Taken together, the positive correlation of ERG-induced mesenchymal-like gene signature in leukemia with solid tumor prostate cancer tissues demonstrate that an ERG-specific gene signature is induced, which is independent of disease origin, and may provide sensitive molecular targets of prognostic value, thereby aiding in predicting the outcome or response to therapy.

### ERG overexpression results in a profound apoptotic resistance

**Figure 2:**
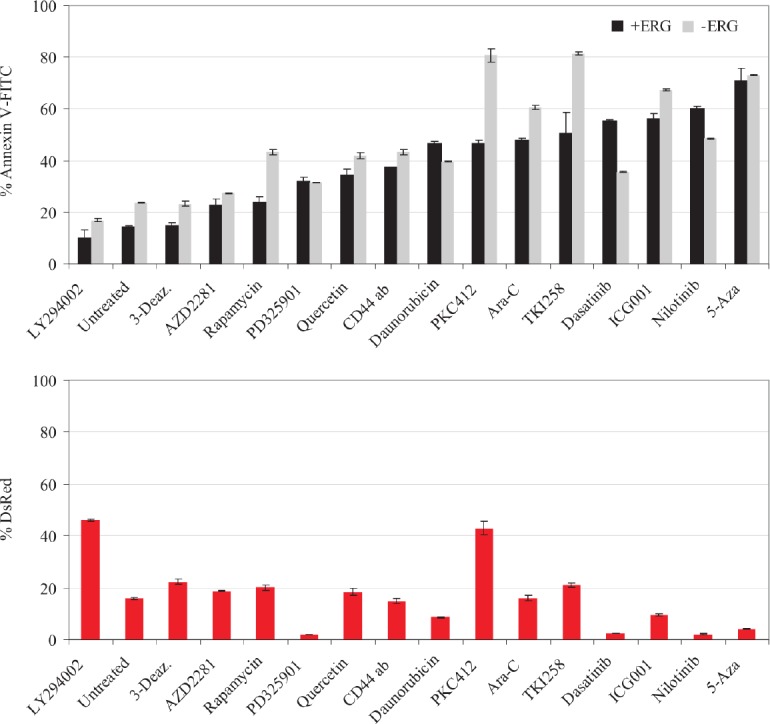
Drugs testing various branches of ERG targeted pathways elicit ERG-mediated drug resistance, which is enhanced by the presence of HS-5 and AML MSCs A panel of 15 drugs (Table 2) was surveyed to determine the extent of ERG dependent drug resistance. ERG-induced and non-induced cells were treated with 15 drug candidates aimed at inducing apoptosis and traced by Annexin V-FITC. Values plotted are the total Annexin V-FITC positive cell population of ERG-induced (+ERG, black bars) and non-induced cells (−ERG, grey bars). Upper plot, ranking by ERG resistance to apoptosis; lower plot, ERG-induced cells (non-apoptotic) were tracked by DsRed fluorescence (+ERG, red bars). Representative results for the CL9 clone are plotted. The error bars denote the standard deviation

Recently, we reported that ERG overexpression conferred resistance to the multi-kinase inhibitors TKI258 and sorafinib[26]. A panel of 15 drugs was selected to further explore the ERG-driven drug resistance. From our own studies in leukemia cells and previous reports in prostate cancer genes, we selected the ERG-targeted networks to include: WNT signaling[21], AKT/PI3K signalling[26,33], ERK[34], DNA repair[35], and DNA chromatin remodeling (Table 2)[36]. ERG-induced drug resistance was determined by three criteria: 1) the significant difference in apoptotic induction in non-induced cells in comparison with ERG-induced cells, 2) the maintenance or expansion of ERG-induced cells (DsRed positive and absence of apoptosis), 3) by the persistence of spindle shape cells upon drug treatment (Table 2). Under those conditions, ERG-induced cells were considered resistant to the following agents: LY294002, rapamycin, quercetin, PKC412, cytarabine (Ara-C), ICG-001, and TKI258 as previously determined[26] (Figure 2A, upper panel). Remarkably, ERG-induced cells were not only resilient to apoptosis when treated with Ara-C, LY294002, or PKC412 but also displayed sustained DsRed population upon Ara-C treatment and expansion of the DsRed population upon PKC412 or LY294002 treatment (Figure 2A, lower panel). Moreover, treatment with AKT inhibitor, LY294002, or PKC inhibitor, PKC412 enhanced spindle shape formation (Table 2). Conversely, ERG-induced cells were sensitive to daunorubicin, dasatinib, nilotinib, and 5-azacytidine as assessed from the diminishing DsRed positive population, and increasing Annexin V-FITC population. The extent of cell death upon PD325901 treatment between ERG-induced and non-induced cells did not differ; however given that the DsRed positive population was depleted, ERG-induced cells were considered sensitive to PD325901. Drugs that did affect apoptosis in both ERG-induced and non-induced cells were 3-deazaneplanocin A and olaparib (AZD2281). The lack of 3-deazaneplanocin A (EZH2 inhibitor)[36] and AZD2281 (PARP inhibitor)[37] effectiveness in ERG-induced cells may be due to repression of the drug targets EZH2 and CHEK1. We conclude that ERG-induced cells are resistant to Ara-C, WNT inhibitors, drugs targeting the protein kinase C pathway (PKC412) and downstream effectors: i.e. the PI3K inhibitors LY294002 and rapamycin. Thus, PKC kinase inhibitors, WNT inhibitors and Ara-C are predicted not to be effective in clearing ERG overexpressing cells in leukemia. By contrast, nilotinib is expected to reverse drug resistance, by selectively enhancing apoptosis most potently in cell model system overexpressing ERG.

**Table 2:** ERG transcriptional pathways are targeted with specific compounds to determine the resiliency of ERG dependent spindle shape formation

**Compound**	**Category**	**Mode of Action**	**Spindle Shape Formation**
Cytarabine (Ara-C)	DNA modulation	incorporation into DNA, inhibition of DNA polymerase	++
Daunorubicin		interacts with DNA by interchalation	−
Olaparib (AZD2281)		inhibiotor of PARP	−
3-deazaneplauocin		inhibitor of EZH2	−
5-Aza-deoxcytidine (5-Aza)		inhibitor of DNA methylation	++
TKI258*	kinase inhibitors	inhibitor of FLT3, c-KIT, FGFR, VEGFR1/2/3, PDGFRß and CSF-1R	+
Nilotinib		inhibitor of BCR-ABL tyrosine kinase	−
Dasatinib		inhibitor of BCR-ABL and SRC tyrosine kinase	−
PKC412		inhibitor of FLT3, PKC, PDGFR VEGFR2	+++
PD325901	ERK inhibitor	inhibitor of ERK	+
Rapamycin	AKT modulation	complexes with FKBP12, indirect inhibitor of mTOR	++
LY294002		inhibitor of PI-3 kinase	+++
6-bromoindirub in-3′-oxime*	WNT modulation	inhibitor of GSK-3b, activation of WNT pathway	−
Quercetin		inhibition of WNT pathway	+
XAV939		inhibition of WNT pathway	++
ICG001		inhibition of WNT pathway	++
CD44	blocking antibody	blocks cell to cell interactions	−

+ indicates <5 adhesive cells/field, ++ indicates <20 adhesive cells/field, +++ indicates >20 adhesive cells/field

− no spindle shape observed

* previous study (Mochmann, et al., 2010 and Bock, et al., 2012)

### ERG overexpressing cells cultured on primary AML-derived stroma have a proliferative growth advantage

Given the survival properties of ERG overexpression, we examined the proliferative properties of ERG-induced cells. CFSE labeled ERG-induced cells displayed a 2-fold proliferative advantage over non-induced cells at 72-hours (Figure 2B). Proliferative growth was further enhanced by PKC412 treatment in ERG-induced cells over non-induced cells, and treatment with Ara-C resulted in minimal advantage over the non-induced group. To further monitor this proliferative advantage, co-cultured ERG-induced cells were harvested in direct contact with the HS-5 stromal layer. Also in this case, ERG-induced cells manifested a 2-fold greater rate of proliferation than cells cultured without stroma (Figure 2B). We further tested the proliferative effect of the direct contact of stroma mesenchymal cells with ERG-induced cells from three AML donors, which confirmed a similar rapid decay rate of CFSE, as observed with the HS-5 co-culture. These results demonstrate a synergistic interaction between ERG overexpressing cells and the contacting HS-5 stroma, or primary AML MSC.

We also investigated ERG-overexpressing cells treated with PKC412, to test the effect of drug resistance in the presence of HS-5 and AML MSCs. Untreated ERG DsRed positive cells were expanded in the presence of HS-5 (10%) but not in the presence of AML MSCs. However, ERG-induced cells (DsRed positive) treated with PKC412 expanded by 40% in co-culture with HS-5 and 20% with AML MSCs (Figure 2C). Thus, ERG-dependent resistance to apoptotic induction was enhanced via stroma protection through cell-to-cell contacts upon PKC412 treatment, in comparison with 80% apoptotic induction in non-induced cells.

### Blocking CD44 by antibody binding inhibits ERG-induced spindle shape morphology

**Figure 3:**
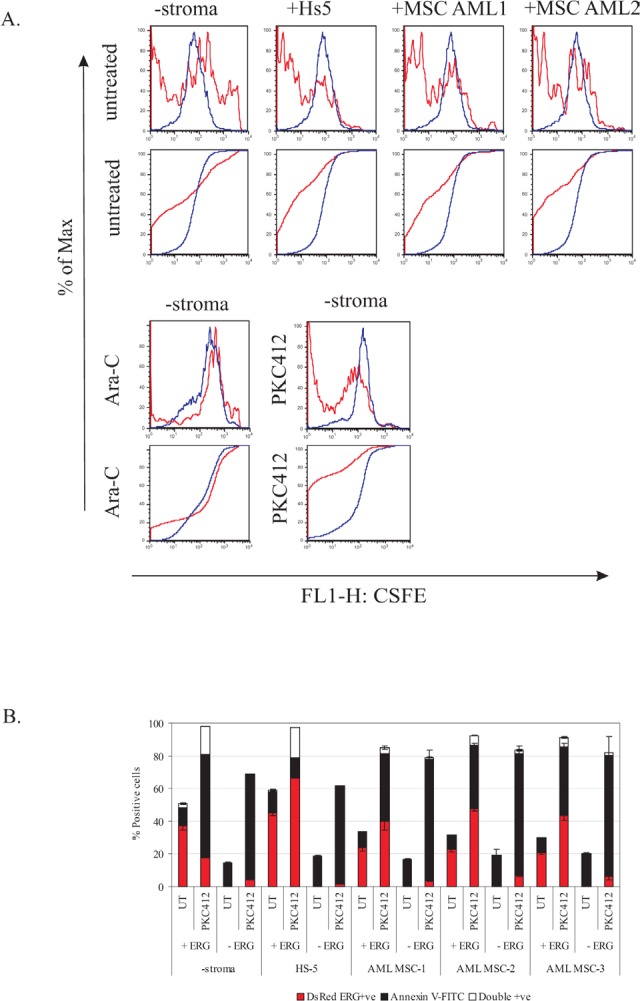
ERG-mediated drug resistance is enhanced by the presence of HS-5 and AML MSCs A) Histograms displaying the decay of CFSE dye as a function of cell division (proliferation) of ERG-induced (+ERG, DsRed positive and CFSE) and non-induced (-ERG, DsRed negative and CFSE) cells. Co-culture in the presence of HS-5 stroma or primary AML-derived MSCs was for 96 hours. Plotted below each histogram is the cumulative distribution function (CDF) plot (black) or the fluorescence distribution (red) for visualization of CFSE decay rates of +ERG versus -ERG cells in the presence of HS-5, AML MSCs, or treatment with Ara-C or PKC412. B) ERG-inducible (+ERG) and non-inducible (−ERG) cells were treated with PKC412 (72 hours) in the presence of either HS-5 stroma or 3 primary AML MSCs. Apoptosis in response to PKC412 was measured with Annexin-V-FITC. ERG-induced cells were traced by DsRed (red bars); ERG-induced apoptotic cells were double positive (DsRed and Annexin V-FITC; white bars), whereas non-induced apoptotic cells were only positive for Annexin V-FITC (black bars). UT, untreated cells processed in parallel. The drug screen, CFSE, and stroma co-culture experiments were conducted in duplicates for both stably transfected K562 pTRE-ERG CL9 and CL46 clones. Representative results for the CL9 clone are plotted in A) and B). The error bars denote the standard deviation.

Due to the enhanced survival of ERG-induced cells, we sought to identify potential adhesion molecules induced by ERG overexpression that would regulate cell-to-cell contacts. A key regulator of mesenchymal-like properties is CD44, a major adhesion molecule for homing, cell migration, and signal transduction[18]. Deregulated CD44 contributes to leukemogenesis, and blocking CD44-mediated adhesion by complexing antibodies to aid in the detachment of leukemia cells from the bone marrow niche has been proposed as a targeted therapy in leukemia[38]. CD44 was significantly over-expressed (2-fold) in the ERG-induced clones. Therefore, we tested a CD44 blocking antibody in culture with ERG-induced and non-induced cells (Figure 3A and 3B). Addition of a CD44 blocking antibody completely abrogated the spindle shape formation in ERG-induced cells, whereas IgG alone did not elicit any morphological changes. ERG-induced cells were then treated with PKC412 to enhance spindle shape formation. In this case, antibody blocking of CD44 inhibited spindle shape formation and reduced cell adhesion by 75%, whereas IgG antibody had no effect. Thus, ERG-induced spindle shape formation is mediated by CD44. We also measured apoptosis to determine the effect of CD44 block on ERG-induced cells. Only marginal changes in apoptosis (Figure 2A) were observed, affirming that CD44's main function is to aid cell-to-cell adhesion of in ERG-induced cells. Thus, disruption of ERG-mediated adhesion via a CD44 block may be of therapeutic significance in combination with current treatment protocols.

### Overexpression of ERG involves ERK 1/2 phosphorylation

**Figure 4:**
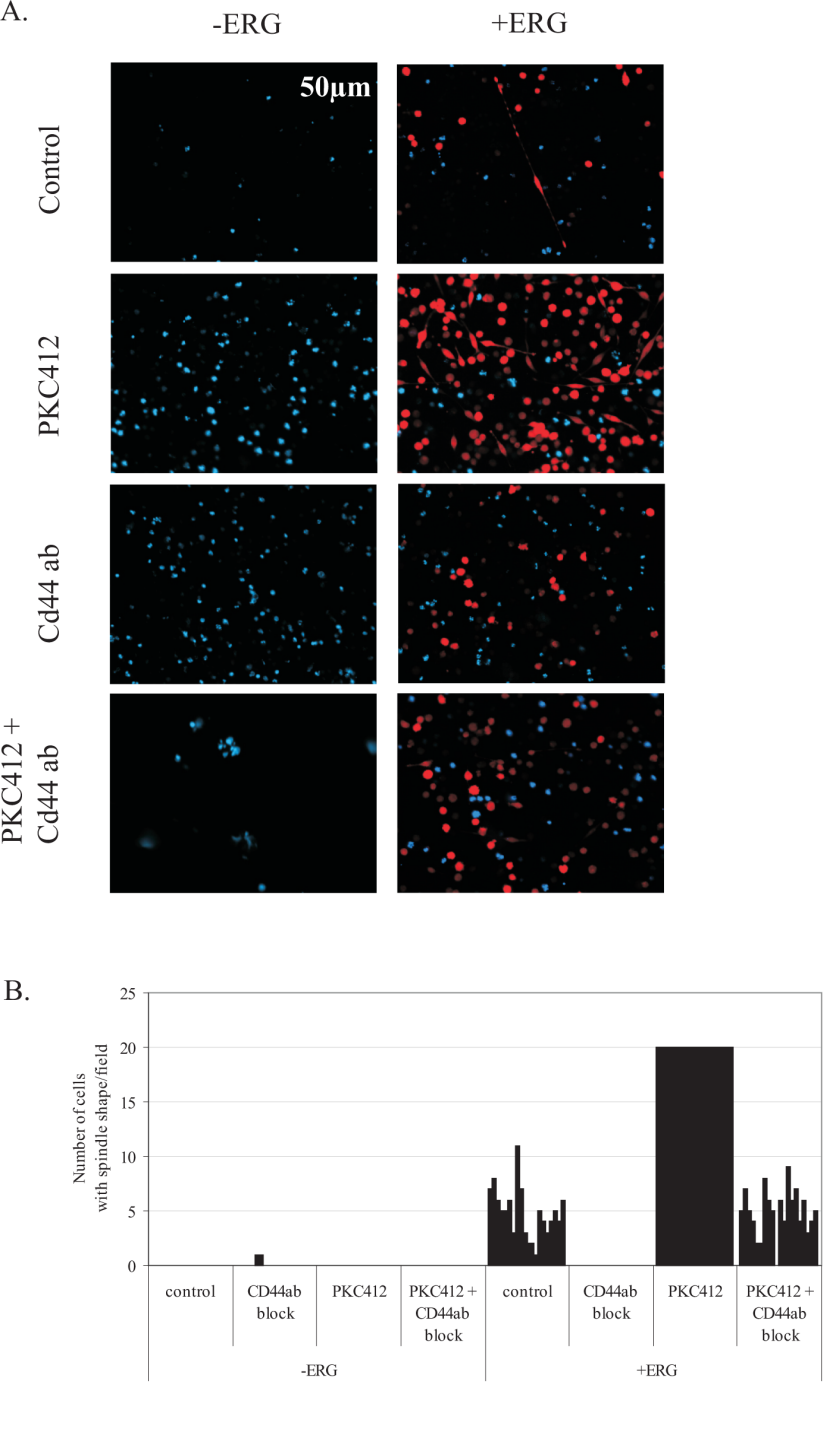
Spindle shape formation induced by ERG overexpression is inhibited by a CD44 blocking antibody A) Representative fluorescence images data of K562 leukemia ERG-induced (+ERG) and non-induced (−ERG) cells after treatment for 72 hours with PKC412, blocking anti-CD44 (1:500 dilution) antibody alone, or the combined PKC412 and anti-CD44 (1:500 dilution) antibody. ERG-induced cells express DsRed and their nuclei were stained with DAPI. B) Graph of cells (counts) developing spindle shape formation for ERG-induced (+ERG) and non-induced cells (−ERG). ERG-induced and non-induced cells were treated with PKC412 to promote ERG dependent spindle-shape formation. The combination of ERG induction and PKC412 treatment induced potent spindle shaped cells in numbers greater than 20/field, which exceeds the error probability of an accurate cell count number. Thus, an upper limit of 20 spindle shaped cells/field was set. The graph represents the results of one of two experiments.

Current evidence supports the notion that, in cancer. MET (as opposed to EMT) is turned on in malignant cells to promote cell proliferation, migration, and evasion from apoptosis[18]. Genome-wide studies demonstrated that ERG activates the RAS/ERK pathway in a transgenic ERG leukemic model[ll], and regulates cell migration in prostate cells[34]. Therefore, we sought to determine if ERG overexpression affected the phosphorylation levels of ERK1/2. Potent ERG overexpression induced higher pan ERK levels compared to non-induced cells, and the effect was enhanced with PKC412 and Ara-C treatment (Figure 4). Endogenous levels of phosphorylated ERK1/2 in ERG-induced cells were also elevated (including with Ara-C treatment), whereas no differences were observed in cells treated with PKC412. The lack of phosphorylation changes in ERK1/2 in cells treated with PKC412 suggests that other residues may be phosphorylated to enhance ERK signal transduction in ERG-induced cells. The upregulated levels of pan and phosphorylated ERK1/2 in ERG-induced cells indicate that signaling is upstream leading to adhesion and resistance to PKC412 and Ara-C treatment.

### Overexpression of ERG generates genomic instability

**Figure 5:**
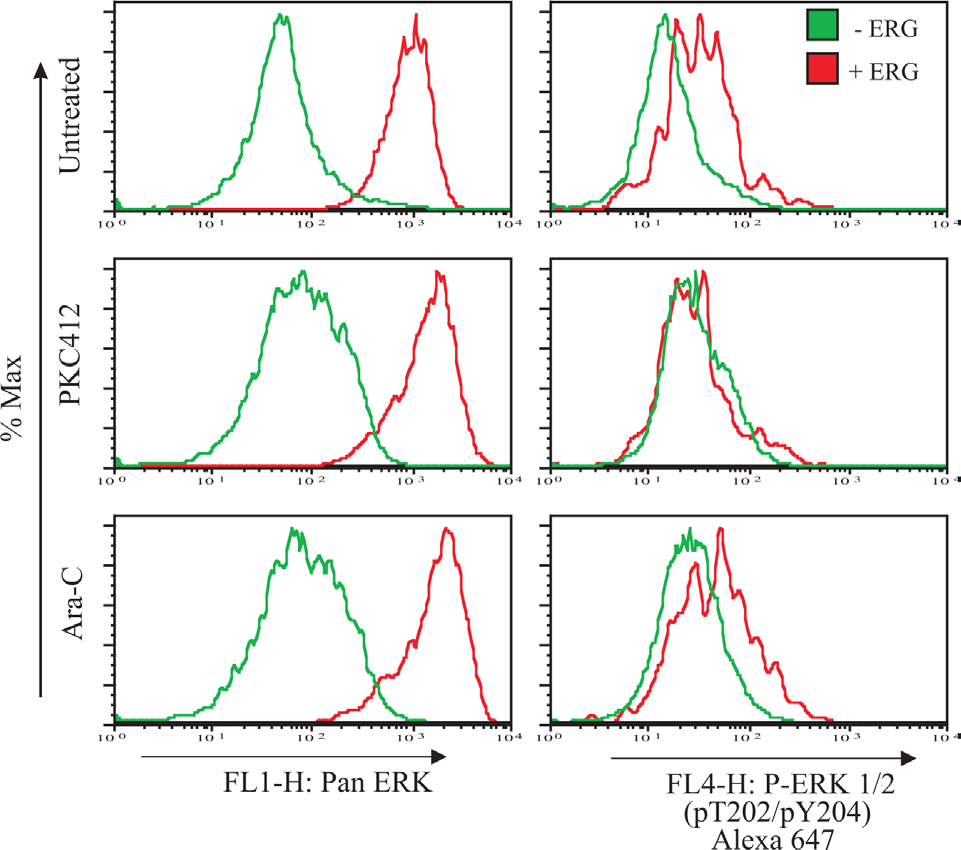
Intracellular staining of pan and phosphorylated ERK1/2 levels increase in cells with ERG overexpression ERG-induced (+ERG) and non-induced (−ERG) cells were probed for intracellular levels of pan ERK1/2 and P-ERK1/2 (pT202/p204), left and right panels, respectively. To determine the extent of ERK activation, ERG-induced (+ERG) and non-induced cells (−ERG) were treated with either PKC412 or Ara-C for 24 hours. Plotted is a representative graph for intracellular probing for pan and phosphorylated levels of ERK1/2 and conducted in duplicate and duplicate in K562 pTRE-ERG clones, CL9 and CL46 clones.

Due to the severe downregulation of DNA repair gene expression in ERG overexpressing cells (Table 1) and in prostate cancer studies[14], we examined ERG overexpressing cells for the presence of DSBs by performing a neutral comet assay[24], with and without Ara-C or PKC412 treatments. The percentage of DNA in the comet tail reflects the number of DSBs. Non-induced cells displayed, on average, 30% tail DNA, whereas ERG-induced cells harbored a significant greater amount (45–50%) (Figure 5). Treatment of non-induced cells with Ara-C or PKC412 increased the percent tail DNA, as expected, to 50% compared to untreated controls. However, ERG-induced cells treated with the cytotoxic drugs showed a significant reduction of DSBs (10% tail DNA with Ara-C and 30% with PKC412). We attribute the reduction of % tail DNA in ERG-induced cells treated with PKC412 to the activation of alternate DNA repair mechanisms, such as non-homologous end-joining, possibly to sustain high DNA replication rates (proliferative growth). Taken together, our data indicate that upon ERG induction, the oncogene generates increased DNA damage (DSBs) and that the effect may be reversed by treatment with Ara-C and PKC412. These observations complement the observed expansion of DsRed in ERG-induced cells and the enhanced proliferative rate in the presence of PKC412 (Figure 2A and B). Hence, aberrant DNA repair may contribute to the multifactorial drug-mediated resistance in high ERG expressers in leukemia.

## DISCUSSION

Herein we show that in leukemia cells ERG overexpression promotes a mesenchymal-like gene expression signature that includes: CD24, CD44, ITGA10, ITGB5, ITGB3, ITGA2B and the morphogenic-associated genes, such as SHANK3, TYROBP. These results are in line with the positive correlation of ERG-induced mesenchymal-like gene signature in leukemia with prostate carcinoma and chemotherapy-treated invasive breast tissues that validated our model system. We propose that, irrespective of cell type, high ERG expressing cells may initiate a malignant phenotype by promoting adhesion thereby enhancing the likelihood of escaping chemotherapeutic agents.

Through fluorescent tracking, we have also demonstrated that ERG overexpression in leukemia cells confers proliferative growth advantage at twice the rate of non-induced cells, whilst mediating the development of spindle shape morphology. In our stable phenotypic model of ERG overexpression, these proliferative features allowed for an even greater ERG-induced cell expansion in co-culture with the cell line HS-5 and in co-culture with primary bone marrow derived stroma from AML. Thus, enhanced adhesion of ERG overexpressing cells to the stromal bed may exploit direct interactions with stroma cells in order to increase cell survival. In the presence of cytotoxic drugs, the percentage of ERG-induced cells treated with PKC412 was enhanced, although the rates of proliferation were not altered (data not shown). In addition the drug resistance promoted directly by ERG, stroma may provide additional apoptotic protection from cytotoxic drugs.

**Figure 6:**
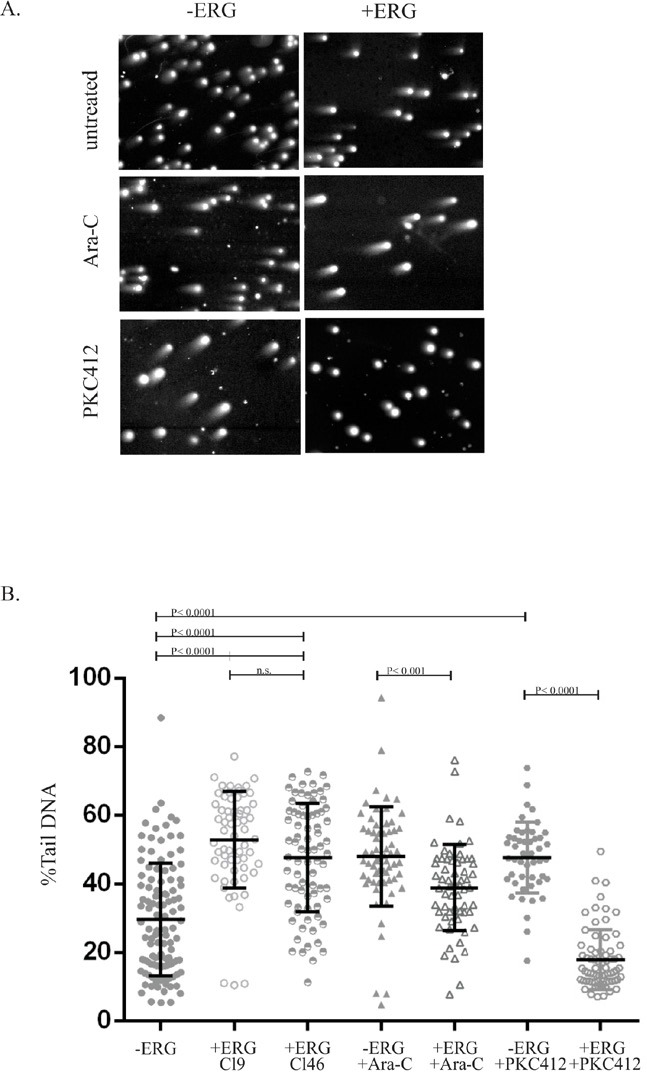
ERG-induced cells result in increased genomic instability and enhanced DNA repair in the presence of Ara-C and PKC412 ERG-induced (+ERG) and non-induced (−ERG) cells were treated with PKC412 or Ara-C for 96-hours and then analyzed by the neutral comet assay to detect the extent of DNA double strand breaks. A) Representative agarose smears of ERG-induced and non-induced cells stained with DAPI in the presence of Ara-C or PKC412. B) Quantification of %Tail DNA was determined following treatment with Ara-C and PKC412 for 96-hours in ERG-induced (+ERG) and non-induced (−ERG) cells. The %Tail DNA was determined for non-induced cells (n=110), ERG-induced cells (CL9 and CL46, n=63 and n=78, respectively), non-induced cells treated with Ara-C (n=63), ERG-induced cells treated with Ara-C (n=62), non-induced cells treated with PKC412 (n=48), and ERG-induced cells treated with PKC412 (n=63). Statistical tests were performed using oneway ANOVA (Tukey test for multiple comparisons).

ERG-induced leukemia cells treated with other PKC or WNT targeting drugs, such as LY294002, rapamycin and ICG-001, promoted expansion of ERG-induced cells rather than inducing apoptosis. This indicates that apoptotic evasion is another feature of ERG-induced survival, particularly under cytotoxic stress, possibly through the inhibition of PKC or WNT signaling pathways. Hence, it seems unlikely that targeted therapy through these drugs will benefit high ERG expressers. Conversely, nilotinib, PD325901, 5-azacytidine, dasatanib, were effective at depleting DsRed ERG positive cells. Of the agents screened, nilotinib induced apoptosis most effectively in ERG-induced cells.

Our treatment with a CD44 blocking antibody completely abrogated the spindle shape phenotype, with minimal apoptotic effects. Due to antigen specificity, blocking monoclonal antibodies have proven to be effective when combined with chemotherapy, such as rituximab for B-Cell lymphoma, trastuzumab for HER2-positive cancer, cetuximab for colorectal carcinoma, and alemtuzumab for chronic lymphocytic leukemia. CD44, in particular, is believed to prevent homing of leukemia stem cells to the bone marrow, thus representing an attractive target for AML therapy[38]. This approach may also be applicable to the subgroup of high ERG expressers in AML in conjunction with other effective drug therapies, such as nilotinib.

The acquisition of drug resistance though activation of ERK1/2 is a common feature of oncogenes. ERK1/2 and downstream targets are regulated by ERG overexpression in the normal prostate cell line RWPE-1 through the ERG target PLAU[34]. Our results concur with these studies, since ERG overexpression resulted in greater amounts of phosphorylated ERK1/2 than in non-induced cells. ERG-induced cells treated with PKC412 led to an increase of pan ERK levels. Surprisingly, phosphorylated levels of pT202/Y204 were not detected, despite the remarkable expansion of ERG-induced cells and the enhanced spindle morphology. Thus, it is possible that either shunting to a different pathway branch occurred, or that phosphorylation took place at different ERK residues that were not recognized by the Y202/T204 antibody. That activation of the ERK1/2 pathway constitutes a critical step in the acquisition of drug resistance in ERG-induced cells is upstream, is further supported by our data with Ara-C, which revealed diminishing levels of ERK1/2 phosphorylation.

In prostate cancer, genomic mutations of DNA repair-associated genes, such as polymerase iota, were positively correlated with TMPRSS2-ERG positive tumors[39], which associates ERG overexpression with the DNA damage response. In ERG positive cells, the DNA repair genes PARP and DNA-PKs were also reported to co-immunoprecipitate with ERG. Likewise, inhibitors of PARP and DNA-PKs were found to impinge on ERG function, since the inhibitor olaparib (AZD2281) had no effect on ERG-mediated cellular proliferation but blocked ERG-mediated cell invasion[37]. In our current study, AZD2281 treatment did not affect apoptotic levels in ERG positive cells but blocked spindle shape formation. We also observed increased levels of DSB in ERG-induced leukemia cells, as previously reported in prostate cancer cell lines[14]. Also, in our leukemia model, ERG under-expressed genes included ATM, CHEK1 and ATR, which are key genes in the initiation of homology-dependent DNA repair. Surprisingly, addition of PKC412 elicited a remarkable lessening in genomic instability in ERG-induced cells, suggesting either a restoration in DNA repair gene expression or a shift from homologous recombination to other types of DNA repair, including non homologous end joining (NHEJ). The latter hypothesis is supported by ERG-induced XRCC5, a key NHEJ-associated gene and significantly ranked with high ERG levels in prostate mRNA expression datasets (Supplement Figure 1A and 1C). Hence, it is possible that a shift from homologous recombination to a NHEJ pathway of DNA repair may contribute to the escape from apoptosis and drug resistance. Further studies would be required to determine the precise repair processes coordinated by ERG in leukemia.

In conclusion, our K562 ERG-induced cell model system unraveled an ERG mesenchymal-like gene signature that overrides disease entities and reflects a general pattern of ERG-driven malignancies. We highlight, a number of changes in gene transcription in key pathways responsible for drug resistance, and provide novel targets for further validation and development in order to achieve custom tailored treatment strategies for the high ERG subgroup of leukemia.

## MATERIALS AND METHODS

### Cells and drug treatment

Cultures of K562 pTRE-ERG doxycycline (DOX) inducible clones were grown in 10% FCS RPMI and seeded at 1×105 cells/ml, as previously described[21]. K562 pTRE-ERG cells were stimulated with DOX (1 μg/ml) for 24 hours. Following this step, drug was administered in the presence and absence of DOX in two K562 pTRE-ERG clones (CL9 and CL46) for an additional 96 hours. Drug (company, concentration ranges) used in this study are as follows: TKI258 (Novartis, 10 μM), PKC412 (LC Laboratories, 10 μM), rapamycin (LC Laboratories, 10 μM), cytarabine (Cell Pharm, 20 μg/ml), quercetin (Sigma, 10 μM), PD0325901 (Selleck Chemicals, 10 μM), daunorubicin (Pfizer, 10 nM), nilotinib and dasatinib (Bug laboratory, Frankfurt Germany, 10 nM), olaparib (AZD22811, Selleck Chemicals, 10 μM), LY294002 (Cayman Chemical, 10 μM), 3-deazaneplanocin A (Cayman Chemical, 10 μM), ICG001 (Selleck Chemical, 10 μM), XAV393 (Sigma, 10 μM), and 5-azacytidine (Sigma, 1 μM). Dose curves were determined by Water Soluable Tetrazolium salt (WST) cell viability assays to determine near IC50 concentrations for each drug in K562 cells.

For co-culture experiments, the HS-5 stroma original cell line was purchased from ATCC and grown in 20% FCS RPMI to 80% confluence. Stromal layers were seeded one day prior to co-culture. ERG-inducible cells were stimulated for an additional 24 hours with DOX and combined with HS-5 stroma in 10% FCS RPMI for an additional 24 hour period before the addition of drugs. ERG-inducible cells and stroma were then co-cultured for an additional 4 days with and without DOX to allow cell morphology changes to take place.

For the counting of spindle shaped cells, K562 pTRE-ERG CL9 and CL46 cells were seeded onto culture dishes with 2 mm grid plates and stimulated with DOX for six days. Two independent experiments with blind labels in duplicate were performed for each experimental group. Rabbit monoclonal anti-CD44 (H-CAM, Millipore) and anti-mouse IgG (Cell Signaling) were used in cell adhesion experiments at 1:500 dilution.

### Microarray expression and bioinformatic analyses

Three independent stably transfected clones (CL4, CL9, CL46) of K562 pTRE-ERG cells were grown in the presence and absence of DOX (ERG-induced and non-induced) for long-term culture (8 days). Total RNA was extracted from ERG-induced and non-induced cells and hybridized using Affymetrix U133-plus chip. Data analysis was performed with Gene Spring 4.2 (Agilent Technologies). Differential expression of genes from biological triplicates (CL4, CL9, CL46) that displayed an over- and under-expression of 2-fold or greater between ERG-induced and non-induced cells were used for further analysis. Microarray data have been deposited in the Gene Expression Omnibus Database. To determine enriched biological terms, over- and under-expressed candidate gene lists were uploaded to the DAVID Bioinformatics server (http://david.abcc.ncifcrf.gov), which was employed to group and define enriched gene ontology terms[22].

### Comparative gene expression analyses

Differential microarray dataset obtained from clones CL4, CL46, and CL9 (+/− DOX) were uploaded to the Oncomine Cancer Profiling Database (www.oncomine.org). Co-expression of ERG over-expressed and under-expressed genes were compared with leukemia, breast and prostate dataseis which were obtained directly through the Oncomine 3.0 software.

### Quantitative RT PCR

Quantitative RT PCR was conducted with Sybr green master mix (Fermentas) and analyses were conducted as previously described[21]. Relative mRNA expression was plotted as fold changes relative to un-induced cells from two independent pTRE-ERG CL9 and CL46 clones.

### Patient material

To harvest mesenchymal stroma cells, the mononuclear fraction of bone marrow was cultured as previously described[23]. Informed consent of patient material was carried out according to the Declaration of Helsinki.

### Immunofluorescence and flow cytometry

Live ERG-inducible adherent cells were stained with primary rabbit polyclonal anti-WNT11 (Novus Biologicals) and secondary anti-rabbit IgG (Alexa Fluor 488 conjugated) antibodies. Annexin V-FITC (BD Biosciences, San Jose, CA) staining was carried out according to manufacturer's instructions. Carboxyfluorescein diacetate succinimidyl ester, CFSE (Cayman Chemical) was used at 1:400 dilution and staining was conducted according to manufacturer's instructions.

To capture endogenous levels of pan ERK and ERK (pT202/pY204), ERG-induced cells were DOX stimulated for three days followed by 24-hour drug treatment (Ara-C or PKC412). Briefly, for intracellular staining, K562 pTRE-ERG cells (5x105 cells/ml) were fixed with 150 μl Phosflow fix buffer I (BD biosciences) at 37°C, washed with PBS, resuspended in 200 ul cold methanol for 10 min, blocked by PBS with 1% BSA, and labeled with the primary antibody (1:100 dilution) for 15min at 4°C. To detect endogenous ERK levels the a-mouse pan ERK and phospho Alexa a-mouse 647 (pT202/pY204) antibodies were used. When required, secondary antibody, anti-mouse IgG (Alexa Fluor 488 conjugated), was used for labeling. The three antibodies were purchased from Cell Signaling (Davers, MA). The labeled cells were measured by flow cytometry and analysed using Flowjo software. All flow cytometry techniques were conducted on a Becton Dickinson FACSCalibur instrument.

### Comet assay

Double strand DNA breakage was measured through the neutral comet assay[24]. ERG-induced cells were stimulated with DOX for 24 hours prior to the addition of Ara-C or PKC412. Following a 72-hour drug treatment, 1x105 cells were suspended into melted 1% agarose (1:10 dilution) and smeared onto a precoated 1% agarose glass slide. Slides were then submerged into lysis buffer (2.5 M NaCl, 0.1 M EDTA, 10 mM Tris pH7.5, 1% Triton X-100, 1% sodium lauryl sarcosine, 0.5 mg/ml proteinase K) overnight at 4°C. Slides were rinsed in TE buffer, equilibrated in an electrophoresis chamber with neutral electrophoresis buffer for 30 minutes and then electrophoresis was conducted for 25 minutes at 25 volts. Slides were then submerged in distilled water, stained with 4′,6-diamidine-2-phenylindol dihydrochloride (DAPI, 1.4 μM), fixed in 70% ethanol and air dried. Experiments were conducted in duplicate, in both CL9 and CL46 clones, with four to five 100x magnification images for each treatment. The fluorescence intensities of the comet head and comet tail of all cells (63 to 110) in each image were scored. Percent DNA in tail and head: the total fluorescence intensity within the delineated comet tail was divided by the total comet intensity (comet head plus comet tail intensity) and multiplied by 100. Measurements were conducted with the Image J software and Comet Tail analysis plugin (rsbweb.nih.gov/IJ).

## Supplementary Figure and Table




