# Supplementary material for: ERG induces a mesenchymal-like state associated with chemoresistance in leukemia cells
Source: Oncotarget. 2013 Dec 4;5(2):351–62. doi: 10.18632/oncotarget.1449 (PMC3964212; doi:10.18632/oncotarget.1449)

ERG induces a mesenchymal-like state associated with chemoresistance in leukemia cells - Mochmann et al

A. ERG over-expressed genes in Grasso Prostate ERG Rearrangement

Rank	P-value	Fold Change	Gene	Reporter	Gene
1	8.70E-15	12.33	ERG	A_23_P57323	ERG
753	0.001	1.24	KIF5B	A_23_P86403	KIF5B
840	0.002	1.32	NOTCH2NL	A_32_P75695	NOTCH2NL
1587	0.007	1.41	ARHGDIB	A_23_P151075	ARHGDIB
1669	0.008	1.32	PIK3C2A	A_23_P36166	PIK3C2A
1717	0.008	1.35	MED23	A_23_P330999	MED23
2052	0.013	1.14	LSM5	A_23_P93750	LSM5
2239	0.015	1.14	LUC7L3	A_24_P382113	LUC7L3
2337	0.017	1.15	CUL2	A_24_P252794	CUL2
2572	0.021	1.14	MED28	A_24_P932632	MED28
2663	0.022	1.26	ZNF107	A_23_P45087	ZNF107
2820	0.026	1.29	PTBP2	A_32_P174832	PTBP2
2908	0.028	1.12	XRCC5	A_24_P345498	XRCC5
3252	0.037	1.22	CLK4	A_24_P123521	CLK4
3311	0.039	1.12	VAPA	A_23_P207957	VAPA
3368	0.040	1.25	ZNF507	A_24_P185487	ZNF507
3443	0.042	1.35	LIG4	A_23_P25581	LIG4
3536	0.044	1.39	ZNF519	A_23_P328766	ZNF519
3589	0.046	1.25	HOOK3	A_32_P49211	HOOK3
3691	0.049	1.16	STAG1	A_23_P357365	STAG1

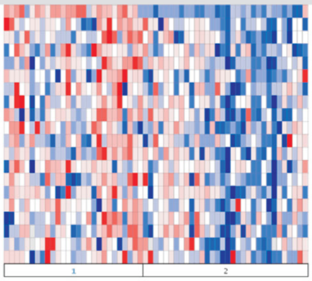

Legend: 1. ERG Rearrangement (27) 2. No ERG Rearrangement (32)

C. ERG over-expressed genes in Stickeler Breast Clinical Specimen

Rank	P-value	Fold Change	Gene	Reporter	Gene
62	4.12E-7	3.18	ERG	A_23_P57323	ERG
240	7.92E-6	3.08	DST	A_23_P348159	DST
312	1.77E-5	2.35	TIMP3	A_23_P399078	TIMP3
383	2.81E-5	2.42	CLU	A_32_P224498	CLU
402	3.34E-5	2.14	TCF4	A_24_P918527	TCF4
470	5.41E-5	3.39	CMTM5	A_23_P106042	CMTM5
585	1.05E-4	2.99	LIFR	A_24_P325992	LIFR
608	1.18E-4	2.39	ITGB5	A_23_P166633	ITGB5
660	1.51E-4	2.33	FLT4	A_23_P356070	FLT4
848	3.13E-4	1.71	PECAM1	A_23_P252471	PECAM1
955	4.54E-4	3.00	MYLIP	A_23_P31041	MYLIP
1069	6.34E-4	2.39	ALDOC	A_23_P78108	ALDOC
1288	0.001	1.79	CLIP1	A_24_P306425	CLIP1
1461	0.002	2.04	ITGBL1	A_24_P916965	ITGBL1
1689	0.003	1.49	USP53	A_32_P128701	USP53
1692	0.003	1.66	ITGB1BP1	A_23_P154507	ITGB1BP1
1702	0.003	1.36	VAPA	A_23_P207957	VAPA
1975	0.004	1.39	MLL5	A_24_P196704	MLL5
2431	0.007	1.43	PCM1	A_23_P82950	PCM1
2708	0.009	1.66	MLL3	A_24_P204269	MLL3

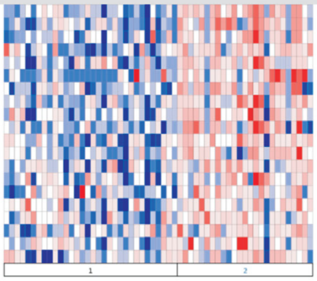

Legend: 1. No Treatment Control (32) 2. Epirubicin/Cyclophosphamide + Docetaxel Treatment (25)

B.ERG over-expressed genes in Grasso Prostate Carcinoma

Rank	P-value	Fold Change	Gene	Reporter	Gene
112	2.16E-10	1.38	LEO1	A_23_P314222	LEO1
304	4.09E-8	3.42	ERG	A_23_P57323	ERG
334	7.52E-8	1.97	CD2AP	A_24_P329152	CD2AP
433	3.40E-7	1.53	MYLIP	A_24_P917123	MYLIP
708	4.57E-6	3.07	ITGBL1	A_24_P916965	ITGBL1
731	5.24E-6	1.47	ACADSB	A_32_P31945	ACADSB
867	1.20E-5	1.43	SGMS2	A_23_P326204	SGMS2
979	2.25E-5	2.26	ASPM	A_23_P52017	ASPM
1050	3.08E-5	1.81	PICALM	A_32_P89099	PICALM
1340	1.05E-4	1.62	HIST1H2BI	A_23_P111041	HIST1H2BI
1617	2.59E-4	1.21	MED23	A_23_P145501	MED23
2098	8.84E-4	1.51	ATP2C1	A_24_P137376	ATP2C1
2210	0.001	2.87	CXCL11	A_23_P125278	CXCL11
2358	0.002	1.19	DCLRE1C	A_23_P86635	DCLRE1C
2427	0.002	1.13	XRCC5	A_24_P345498	XRCC5
2880	0.004	1.16	FCER1G	A_23_P160849	FCER1G
2962	0.005	1.12	LSM5	A_23_P93750	LSM5
3330	0.008	1.28	GP1BA	A_24_P270829	GP1BA
3360	0.008	1.29	ARHGDIB	A_23_P151075	ARHGDIB
3433	0.009	1.38	ZNF37A	A_24_P239668	ZNF37A

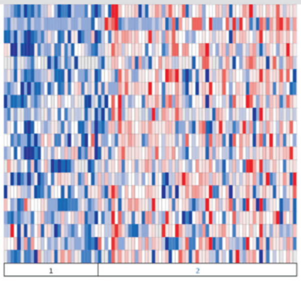

Legend:1. Prostate Gland (28) 2. Prostate Carcinoma (59)

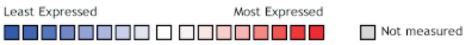

Supplement: Supplementary file 1 [file oncotarget-05-351-s001.pdf]
