# Supplementary material for: ERG induces a mesenchymal-like state associated with chemoresistance in leukemia cells
Source: Oncotarget. 2013 Dec 4;5(2):351–62. doi: 10.18632/oncotarget.1449 (PMC3964212; doi:10.18632/oncotarget.1449)
Supplement: Supplementary file 2 [file oncotarget-05-351-s002.pdf]

ERG induces a mesenchymal-like state associated with chemoresistance in leukemia cells - Mochmann et al

Supplement Table 1. Source and significance of *ERG* induced genes mined with other cancer datasets. Oncomine™ (Compendia Bioscience, Ann Arbor, MI) was used for analysis and visualization.

Dataset	Source	Disease	Comparison	P-Value	Fold change
Grasso Prostate	Nature 2012	Prostate cancer	ERG rearrangement versus no rearrangement	8.70E-15	12.3
Grasso Prostate	Nature 2012	Prostate cancer	Prostate carcinoma versus prostate gland	8.71E-08	3.4
Stickeler Breast	Oncol Rep 2011	Breast cancer	Epirubicin/Cyclophosphamide + Docetaxel Treatment versus no treatment	4.10E-07	3.18
